# Efficacy of Pre-Medication with Ibuprofen on Post-Operative Pain after Pulpotomy in Primary Molars

**DOI:** 10.22037/iej.v13i2.16624

**Published:** 2018

**Authors:** Lili Shafie, Sara Esmaili, Masoud Parirokh, Abbas Pardakhti, Nouzar Nakhaee, Paul V. Abbott, Hamide Barghi

**Affiliations:** a *Pedodontist, Fellowship in Sedation and Hospital Dentistry, Kerman, Iran; *; b * Department of Pediatric Dentistry, Dental School, Hormozgan University of Medical Sciences, Bandar Abbas, Iran; *; c * Endodontology Research Center, Dental School, Kerman University of Medical Sciences, Kerman, Iran; *; d * Pharmaceutics Research Center, Neuropharmacology Institute, Kerman University of Medical Sciences, Kerman, Iran; *; e * Neuroscience Research Center, Kerman University of Medical Sciences, Kerman, Iran; *; f * Dental School, University of Western Australia, Perth, Australia; *; g * Department of Pediatric Dentistry, Dental School, Shiraz University of Medical Sciences, Shiraz, Iran*

**Keywords:** Ibuprofen, Pre-Medication, Primary Molar, Pulpotomy, Visual Analogue Scale, Wong Baker

## Abstract

**Introduction:**

Pain management following dental procedures, particularly pulpotomies and extraction, is of great importance in pediatric dentistry. The aim of this study was to investigate the efficacy of pre-treatment with ibuprofen on post-operative pain following pulpotomy of primary molars.

**Methods and Materials:**

In a split mouth double-blinded randomized clinical trial, 49 children aging between 6-10 years old were given either ibuprofen or a placebo 45 min prior to the treatment. After pulpotomy and placement of a stainless steel crown (SSC), the pain level was evaluated using the Wong-Baker face visual analogue scale for up to 7 days post-treatment. McNemar and Wilcoxon tests were used for data analysis.

**Results:**

Forty-five patients were eligible to participate in this study. Pre-medication with ibuprofen significantly reduced pain during the first 24 h post-treatment (*P*=0.032). However, there was no significant difference in the pain levels between placebo and ibuprofen groups at 48 and 72 h post-treatment (*P*=0.154 and *P*=0.197, respectively). The number of times patients needed analgesics in ibuprofen group was significantly lower compared to that in the placebo group (*P*=0.008).

**Conclusion:**

Pre-medication with ibuprofen resulted in less pain following pulpotomy and SSC placement in primary teeth.

## Introduction

One of the main reasons that children find dental procedures unpleasant is pain and discomfort during and after this treatment. The memory of the pain during primary experiences may prevent children from going to the dentist to receive regular check-ups and treatment even years after the first unpleasant experience [1].

It has been reported that 37% to 95% of children may feel pain following various dental procedures, such as tooth extraction or pulpotomy and stainless steel crowns (SSC) [2-7]. Pulpotomy and placing SSC are routine dental procedures performed on a daily basis in pediatric dental practices and in many general dental practices. Therefore, seeking methods to decrease pain and discomfort following such dental procedures is very important [3].

Several studies have reported that pre-treatment with analgesic medications significantly reduces post-operative pain after root canal treatment in adults [8-10]. Ibuprofen, paracetamol, diclofenac and tramadol have been used to manage pain in pediatric dentistry [11-16]. However, paracetamol and ibuprofen are the most commonly used analgesics for post-operative pain in children and have been used in several studies as pre-operative analgesics to investigate their effect on post-operative pain following tooth extraction in children [14, 16]. Although, a systematic review could not confirm their efficacy on post-operative pain when used as pre-emptive medications [17].

Paracetamol and ibuprofen are widely prescribed in children and are the most frequently used over-the-counter analgesics and antipyretics. It has been reported that single doses of ibuprofen (4-10 mg/kg) and paracetamol (7-15 mg/kg) have similar efficacy for relieving moderate to severe pain, and similar safety as analgesics or antipyretics in children. While post treatment prescription of ibuprofen (5-10 mg/kg) is more effective antipyretic than that of paracetamol (10-15 mg/kg) [18]. Most studies investigating the effect of pre-treatment medication on post-operative pain have included extraction of primary molars and none of them have evaluated pain following pulpotomy and SSC placement in primary molars [11-16]. Therefore, the aim of the present study was to evaluate the efficacy of premedication with ibuprofen on post-operative pain following pulpotomy in primary molars.

## Materials and Methods

This split mouth randomized clinical trial was approved by the Ethics Committee of Kerman University of Medical Sciences in Kerman, Iran (Ref No. KA/93/210) and was also registered online (IRCT approval code: IRCT2016102930553N1).

The mean pain score during the first 24 h following treatment was considered the primary outcome. The sample size calculation was based on figures derived from a relevant study in Turkey [16] considering α=5% and power of 80%, to detect a significant 20% difference in pain score (SD=0.9) a sample size of 44 patients was calculated. 

A total number of 49 patients aged between 6 to 10 years old (20 female and 29 male) who had been referred to the postgraduate clinic of the Pediatric Department at Kerman Dental School in Kerman, from October 2013 to June 2014, participated in this study.

The patients’ parents were fully informed about the nature, aim and method of the study and they provided their informed consent on behalf of their children to participate in the study. 

The inclusion criteria were: patients aging 6-10 years old who had bilateral primary molar teeth with carious lesions, the teeth were suitable for restoration, no tenderness to percussion and no pain except after eating, teeth without symptoms, no clinical and/or radiographic signs of pulp degeneration, no sinus tract, no or minimal physiological resorption (less than two-thirds of the root length affected), and there was bleeding of the pulp at the time of cavity preparation and pulp exposure.

Exclusion criteria were: any systemic diseases that contra-indicated conservative pulp therapy, or radiographic findings such as internal resorption, widening of periodontal ligament space, furcation or periapical radiolucency, and root canal calcifications.

In order to carry out a double blinded study, ibuprofen and placebo elixirs were placed in similar single-dose glass bottles. The placebo had the same color, odor and taste as the ibuprofen elixir. The medication dose was calculated based on the patient’s weight. Therefore, each bottle of medication contained 4-10 mg/kg of ibuprofen, while the placebo bottle contained a strawberry-flavored starch suspension. The bottles were encoded and only the pharmacist who prepared the medications was aware of their content. Two bottles containing either ibuprofen or placebo were prepared for each patient and 45 min prior to each appointment, one of the bottles was given to the patient. The operator watched the patient to ensure that the patient drank the entire content of each bottle. 

After administering local anesthesia using 2% lidocaine with 1:80000 epinephrine (Persocaine-E, DaruPakhsh Pharmaceutical Mfg. Co., Tehran, Iran) and placing rubber dam, a pulpotomy was performed using a sterile round bur (Tizkavan, Tehran, Iran) in a high-speed handpiece with copious water irrigation. Any remaining coronal pulp tissue was removed with a sharp spoon excavator. After all the pulp had been removed, the cavity was rinsed with 0.9% normal saline and a wet cotton pellet was applied to obtain hemostasis. If hemostasis was not achieved in both teeth, the patient was excluded from the study and a pulpectomy was performed for the tooth. Calcium-enriched mixture (CEM) cement (Bionique Dent, Tehran, Iran) was used as the pulpotomy agent in the teeth with successful hemostasis. Once the material had set, a restorative glass ionomer cement (GC Corporation, Tokyo, Japan) was placed over the CEM followed by final restoration using a SSC (3M ESPE, Norristown, PA, USA).

All dental procedures including anesthesia, pulpotomy and SSC placement were performed by a second year postgraduate pediatric dentistry student. The patients’ parents were asked to complete a Wong-Baker faces pain rating scale (Wong-Baker FPRS) by asking their children how much pain they had. The pain intensity was rated for up to 7 days post-treatment. The Wong-Baker FPRS consists of a row of 6 numbered faces ranging from “no hurt” (score=0) to “hurts worst” (score=5). The pain severity scoring was outlined as follows: 0; no pain, 1-2; mild pain, 3; moderate pain, and 4-5; severe pain. The parents were also given an elixir of ibuprofen (Neda Pharmacologic Co., Tehran, Iran) after the treatment to be used as an analgesic in case the child had pain requiring relief. They were also asked to record the number of times the analgesic was used. The maximum use of the analgesic was one dose every 6 h and the parents were given an emergency phone number if there was any need for consultation in case of the patient had severe pain.

**Table 1:** Analgesic consumption among patients with and without premedication

**Consumption Time**	Total		Single dose in a day		Double doses in a day	
	Placebo	Ibuprofen	Placebo	Ibuprofen	Placebo	Ibuprofen
****24 h****	17	12	15	12	2	0
****48 h****	8	3	8	3	0	0
****72 h****	3	2	3	2	0	0
****Total****	28	17	26	17	2	0

**Figure 1:**
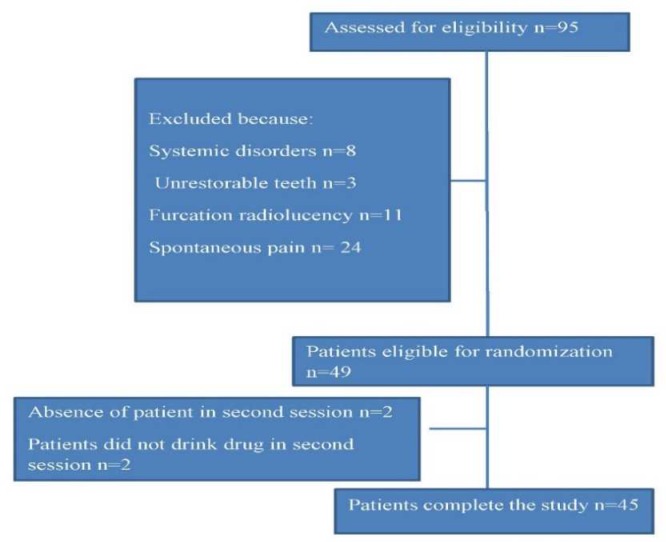
Patient flowchart

Data were analyzed with McNemar and Wilcoxon tests and the level of significance was set at 0.05.

## Results

Forty-nine patients were eligible to participate in this study (Figure 1); however, two patients were excluded since they did not return their forms after the first treatment visit and another two patients were excluded because they did not want to use the medications at the second visit. None of the patients’ parents reported any side effects after taking the medicine. Overall, 17 females and 28 males participated in this study. The average age of the patients was 6.8±0.63 years (range from 6.17 to 7.43). Totally, 19 maxillary and 26 mandibular primary molars were treated. 

**Figure 2:**
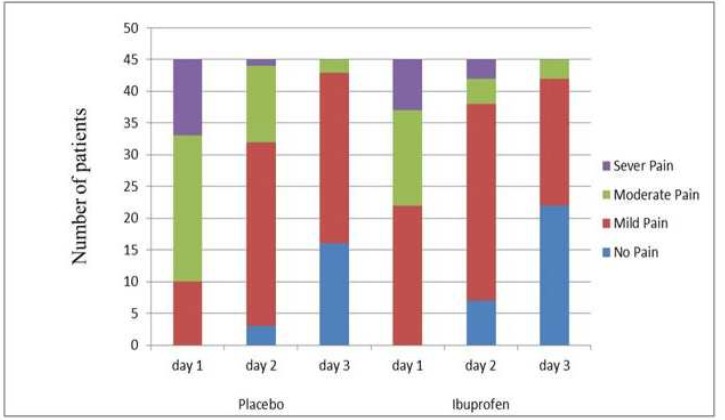
The patients who received ibuprofen pre-operatively reported significantly less pain than the placebo group over the 72 hours post-operative following pulpotomy

**Figure 3:**
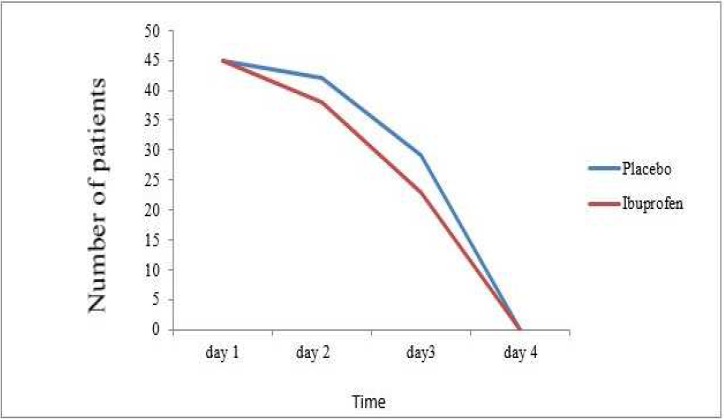
Comparison of pain score of between placebo and ibuprofen over the fourth day post-operative following pulpotomy

None of the patients had pre-operative pain; however, they all reported mild to severe pain after the treatment. The patients who received ibuprofen pre-operatively reported significantly less pain than the placebo group during the first 24 h post-treatment (*P*=0.032) (Figure 2). However, there were no significant differences between the two groups at 48 and 72 h post-treatment (*P*=0.154 and *P*=0.197, respectively) (Figure 2). The patients who received ibuprofen pre-operatively used significantly less analgesics compared to the placebo group (*P*=0.008). Furthermore, the number of the patients who used analgesics in the ibuprofen group (37.8%) was also significantly lower (62.2%) compared to the placebo group (*P*=0.013) (Figure 3 and Table 1). None of the patients reported pain by the fourth day after the procedure (Figure 3).

## Discussion

The results of the present study have shown that pre-treatment with ibuprofen significantly decreased post-operative pain and the need for analgesics during the first 24 h following pulpotomy and SSC placement in primary molar teeth (*P*<0.05).

Calcium silicate cements, such as mineral trioxide aggregate (MTA) and CEM cement, have been used in dentistry for pulp capping, perforation repair, apical plug and root-end filling [19-23]. Both MTA and CEM cement have been successfully used as pulpotomy agents in primary molar teeth [24-26]. In the present study, CEM cement was used as the pulpotomy agent. 

All previous investigations of pain control in children have focused on strategies to manage distress and discomfort following dental extractions, particularly after general anesthesia [14, 27, 28]. Despite some reports regarding the benefits of using pre-operative analgesics to reduce post-operative pain in children [14, 16], two systematic reviews and a meta-analyses have illustrated the lack of evidence-based studies investigating the efficacy of pre-medication with analgesics on post-operative pain in children [17, 29]. 

Previous investigations that compared post-operative pain following extraction of primary molar teeth reported that administration of paracetamol, ibuprofen and diclofenac reduced the pain scores following primary teeth extraction in children [11-16, 28]. Some studies have reported that ibuprofen and paracetamol had equal efficacy on pain relief, whereas others have shown that ibuprofen provided significantly higher pain relief compared to paracetamol [30]. Results of an early systematic review and a meta-analysis showed that ibuprofen and paracetamol had the same analgesic and antipyretic effect [18]. 

However, a more recent meta-analysis has shown that ibuprofen is a more effective pain reliever compared to paracetamol which is in accordance with other study that has previously reported [30]. Paracetamol may be considered by some clinicians to be a safer analgesic than ibuprofen which is a non-steroidal anti-inflammatory drug. However, it has been reported that both medications have similar safety spectra [31].

Therefore, in the present study ibuprofen was used as the pre-medication analgesic. Ibuprofen and paracetamol are among the most widely used analgesic compounds for treating mild to moderate dental pain. However, the results of a multicenter and double blind study in patients with postoperative dental pain has been showed that 400 mg ibuprofen provided superior analgesia over 1000 mg paracetamol that was both clinically and statistically significant. Similar efficacy for relieving moderate to severe pain, and similar safety as analgesics or antipyretics in children has been reported with single doses of ibuprofen (4-10 mg/kg) and paracetamol (7-15 mg/kg) [18]. 

Therefore, in the present study ibuprofen was used as the pre-medication analgesic. Ibuprofen and paracetamol are among the most widely used analgesic compounds for treating mild to moderate dental pain. However, the results of a multicenter and double blind study in patients with postoperative dental pain has been showed that 400 mg ibuprofen provided superior analgesia over 1000 mg paracetamol that was both clinically and statistically significant. Similar efficacy for relieving moderate to severe pain, and similar safety as analgesics or antipyretics in children has been reported with single doses of ibuprofen (4-10 mg/kg) and paracetamol (7-15 mg/kg) [18].

In a preliminary investigation, it has reported that the children pre-treated with an analgesic experienced less pain following various dental procedures [12]. However, they suggested more studies with larger sample sizes and more precise pain evaluation methods should be employed. An investigation has shown that pulpotomy followed by placement of a SSC induced a high level of post-operative pain in children [3]. Therefore, in the present study the same dental procedure was used to evaluate the efficacy of pre-treatment with analgesic on post-operative pain in children. 

In the present study, all children reported pain following the procedure. However, most of them had only mild pain. The reason for the more severe pain in this study compared to previous reports [2-7] can be attributed to the emphasis placed on the patients’ parents to record any pain and discomfort following treatment. The percentage of children who used analgesics once or twice at the day following treatment (37.8% in the ibuprofen group and 62.2% in the control group) showed that the pain was not severe enough; therefore, parents did not need to use analgesics to control their child's pain.

## Conclusion

In conclusion, the present study has shown that pre-medication with ibuprofen significantly reduced the amount of post-operative pain during the first 24 h following pulpotomy and SSC placement. It has also reduced the need for using analgesics post-treatment for children undergoing these procedures.
